# An archipelago within an archipelago: A checklist of liverworts and hornworts of Kepulauan Sunda Kecil (Lesser Sunda Islands), Indonesia and Timor-Leste (East Timor)

**DOI:** 10.3897/phytokeys.180.65836

**Published:** 2021-08-03

**Authors:** Ainun Nadhifah, Lars Söderström, Anders Hagborg, Eka Aditya Putri Iskandar, Ida Haerida, Matt von Konrat

**Affiliations:** 1 Cibodas Botanic Garden, Research Center for Plant Conservation and Botanic Gardens, Indonesian Institute of Sciences (LIPI), West Java, Indonesia Research Center for Plant Conservation and Botanic Gardens, Indonesian Institute of Sciences Cianjur Indonesia; 2 Norwegian University of Science and Technology, Trondheim, Norway Norwegian University of Science and Technology Trondheim Norway; 3 The Field Museum, Chicago, USA The Field Museum Chicago United States of America; 4 Herbarium Bogoriense, Research Center for Biology, Indonesian Institute of Sciences (LIPI), West Java, Indonesia Research Center for Biology, Indonesian Institute of Sciences Bogor Indonesia

**Keywords:** Checklist, hornworts, Indonesia, Kepulauan Nusa Tenggara, Kepulauan Sunda Kecil, Lesser Sunda Islands, liverworts, Timor-Leste (East Timor)

## Abstract

The first ever liverwort and hornwort checklist is provided for the Kepulauan Sunda Kecil (Lesser Sunda Islands) of Indonesia and Timor-Leste (East Timor). We report 129 accepted taxa, 12 doubtful taxa and three rejected taxa previously reported for the Lesser Sunda Islands. The list is based on over 130 literature references, including monographs, regional studies, and molecular investigations. It is clear that bryophytes from this region have been overlooked historically, and under collected, compared to seed plants, birds, and other organisms, forming a remarkable gap in the flora of Indonesia. Publications dealing with liverworts of Lesser Sunda Islands are few and scattered. We predict that further fieldwork, in addition to collections unveiled from regional herbaria, will uncover a number of new records that remain to be reported, especially considering that regionally widespread species have been recorded elsewhere.

## Introduction

**Figure 1.**
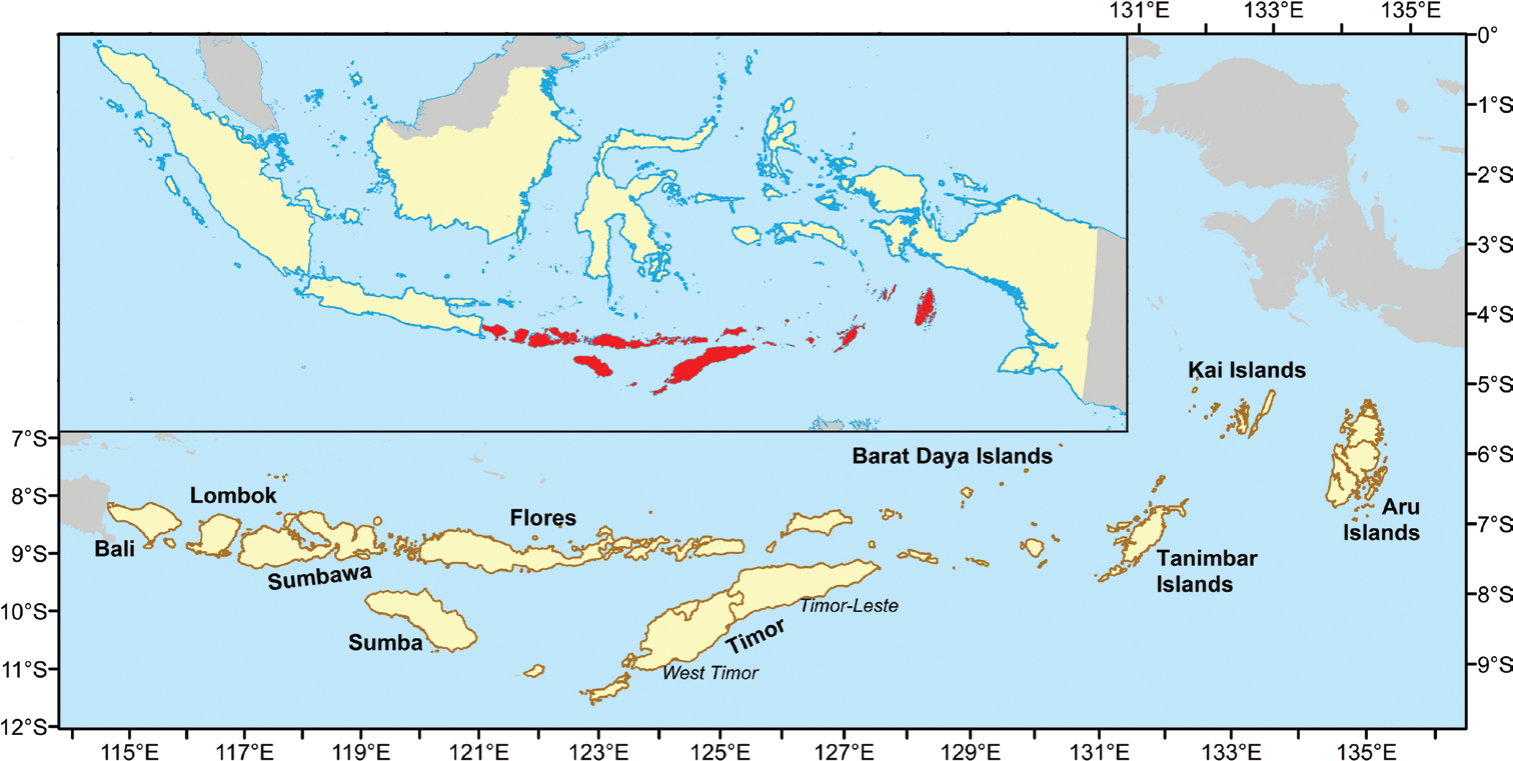
Map of Lesser Sunda Islands with an inset of Indonesia.

The Lesser Sunda Islands (**LSI**), known in Indonesia as Kepulauan Sunda Kecil, are an area covering a longitudinal distance of some 600 kilometres in the southeastern portion of Indonesia, extending between Java in the west and New Guinea in the east (Fig. 1). Lesser Sunda Islands include a multitude of islands, the major ones of which are Flores, Sumba, Sumbawa, and Timor. The region overlaps with two different countries; Indonesia, which includes four different provinces, i.e., Bali, West Nusa Tenggara, East Nusa Tenggara (including Western part of Timor island), and part of Moluccas (van Steenis-Kruseman and van Steenis 1950; Monk et al. 1997; Jepson and Whittaker 2002) and Timor-Leste (East Timor), which includes the eastern part of Timor island (Kusuma 2017). The Lesser Sunda Islands occur as two geologically distinct island chains termed the Inner and Outer Banda Arcs (Audley-Charles 2011). This archipelago also occurs at the heart of the complex crossroads of two continents, Asia and Australia, and two oceans, the Indian and Pacific (Monk et al. 1997). The Lesser Sunda Islands may act as ‘stepping stones’ for animals and plants dispersing between the Greater Sunda Shelf, i.e., the Malay Peninsula, Borneo, Sumatra, Java, and Bali, and the Sahul Shelf, i.e., New Guinea, Australia, and their land-bridge islands (Reilly 2016). Bordered to the west by the Greater Sunda Shelf and to the east by the Sahul Shelf, the Lesser Sunda Islands can be considered oceanic islands in the sense that they have never been connected by land to continental Asia or continental Australo-Papua.

The island chain of LSI comprises 5.2% of endemic species based on families treated in Flora Malesiana (van Welzen et al. 2005). Interestingly, the Indonesian part contains the highest percentages of endemic plants of Indonesia, i.e., 55% (Bappenas 2016). Some important and better-known endemic species in this ecoregion are the Komodo dragon (*Varanuskomodoensis*), the largest lizard in the world and the iconic tree of East Nusa Tenggara Province, sandalwood tree (*Santalumalbum* L.), and *Dracaenamultiflora* Warb. ex Sarasin (Monk et al. 1997).

**Figure 2.**
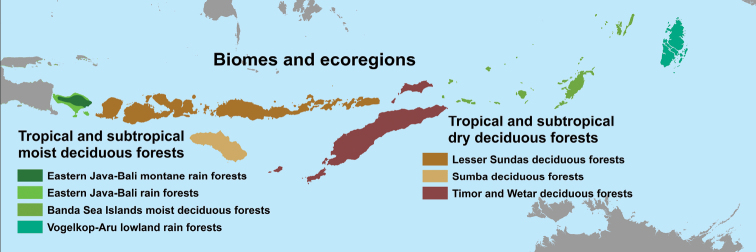
Seven ecoregions in two biomes of Lesser Sunda Islands following the Terrestrial Ecoregions of the World.

The islands of LSI include seven ecoregions in two biomes, Tropical and Subtropical Moist Forests and Tropical and Subtropical Dry Forests (Fig. 2; Terrestrial Ecoregions of the World; Olson et al. 200l). The “Tropical and Subtropical Moist forest” includes two ecoregions on Bali, and two ecoregions on the eastern islands. The Eastern Java-Bali rainforest (https://www.worldwildlife.org/ecoregions/im0113) and Eastern Java-Bali Montane Rain Forest (https://www.worldwildlife.org/ecoregions/im0112) are situated on the Sunda shelf and both are classified as endangered ecoregions since a lot of forest has been cleared. It forms transitional vegetation types relative to the drier areas of the central islands situated on the Sahul shelf.

The “Tropical and Subtropical Dry forests” consist of three ecoregions from Lombok in the west to Timor and Wetar in the east. All three ecoregions are critically endangered/endangered. The largest of the ecoregions is “Lesser Sunda Deciduous Forests” (https://www.worldwildlife.org/ecoregions/aa0201) on Lombok, Sumbawa, Flores and west to Alor including smaller surrounding islands. It consists mainly of semi-evergreen dry forests. With an average annual rainfall of 1,349 mm, this is the driest area but also the most seasonal in Indonesia (Touw 1992). The “Sumba Deciduous Forest” (https://www.worldwildlife.org/ecoregions/aa0203)” was also dominated by deciduous monsoon forest, but now much of it has been replaced by savanna and grasslands (Monk et al. 1997). The “Timor and Wetar Deciduous Forest” (https://www.worldwildlife.org/ecoregions/aa0204) is also largely deforested and replaced by grasslands and savanna.

Further east, two more ecoregions of “Tropical and Subtropical Moist Deciduous Forests” occur. The “Banda Sea Islands Moist Deciduous Forest” (https://www.worldwildlife.org/ecoregions/aa0102) includes all islands west of Timor/Wetar, except Aru Island. It includes evergreen rain forest (Kepulauan Kai), semi-evergreen rain forest, moist deciduous forest, and dry deciduous forest (Monk et al. 1997). The forests are still largely intact, but the ecoregion is still classified as vulnerable. The “Vogelkop-Aru Lowland Rain Forest” (https://www.worldwildlife.org/ecoregions/im0128) is mostly confined to New Guinea but occurs also on Aru. Large parts of the ecoregion are still intact.

Bryophytes, including mosses, liverworts and hornworts, are the second largest group of land plants after flowering plants and are pivotal in our understanding of early land plant evolution (e.g., Ligrone et al. 2012; Zhang et al. 2020). Bryophytes play a significant ecological role including CO_2_ exchange (De Lucia et al. 2003), plant succession (Cremer and Mount 1965), production and phytomass (Frahm 1990), nutrient cycling (Coxson 1992) and water retention (Pócs 1980; Gradstein et al. 2001). Retnowati et al. (2019) cited 849 species of liverworts, 28 species of hornworts and 1,884 species of mosses which are scattered in the major islands of Indonesia. As with many regions in the world, it is evident that the bryophyte, especially the liverwort flora, remains very poorly known in comparison to the vascular flora. Gradstein and Culmsee (2010) noted there are few studies from Southeast Asia investigating the diversity and ecology of tropical bryophytes.

It is clear that bryophytes from this region have been overlooked historically, and under-collected, compared to seed plants, birds, and other organisms, forming a remarkable gap in the flora of Indonesia and/or Timor-Leste. Publications dealing with liverworts of LSI also are few and scattered. The first apparent report of liverworts from LSI was by Sande Lacoste (1856) in the mid-19^th^ century. It was not until the late 19^th^ century and early 20^th^ century that further influential works appeared, including those by Schiffner (1898, 1900, 1955), Stephani (1886, 1899, 1907, 1908, 1909, 1911, 1917, 1924), Verdoorn (1930, 1934a, 1934b, 1935, 1937), and others. Among those islands, Bali is the most explored island (Hegewald and van Zanten 1986; Eggers et al. 1998; Schäfer-Verwimp 2006, 2009; Haerida et al. 2010; Alam 2012; Heinrichs et al. 2012; Girmansyah et al. 2013) with 101 species accepted here. Söderström and Séneca (2008) reported only 61 number of liverworts for Lesser Sunda Islands and considered that this low number of species was the effect of the under-explored areas. More recently, little botanical work has been done in the area; the area has occasionally been visited by students and researchers from nearby institutions, but publications are still lacking. This checklist will complement the survey of mosses of Lesser Sunda Islands by Touw (1992) who enumerated 367 species for the area, and complement other checklists of liverworts from Indonesia, including Java (Söderström et al. 2010), Bali (Haerida 2015, 2017) as well as Sumba (Haerida et al. 2020).

We here present the first-ever checklist of liverworts and hornworts for the Lesser Sunda Islands to serve as the baseline information in our study of the liverworts diversity of this archipelago. Furthermore, this checklist can serve to promote and encourage bryological research in the region. The significance of checklists is summarized by Söderström et al. (2008), including outlining the utility of checklists as powerful and important tools, and their applicability to taxonomy, systematics, and conservation.

**Figure 3.**
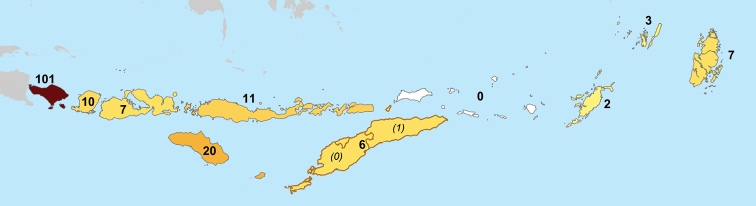
Number of known species from the individual islands. The darker the color, the greater the number of species.

As with many other regions in the world, given the relatively poor focus on liverworts in LSI historically, we predict that a vast number of new records are yet to be reported for the area. In this checklist we report 129 taxa, with another 12 taxa questioned and three rejected. The number of known species from individual islands varies from 101 (Bali) to 0 (Barat Daya Islands), but only Bali has more than 20 known species (Fig. 3).

## Materials and methods

Nomenclature and taxonomy follows the world checklist of hornworts and liverworts (Söderström et al. 2016) with a few updates from recent taxonomic literature. Sources include over 130 publications found through the work of Early Land Plants Today (**ELPT**) database of liverwort taxonomy and distribution, and with some consultation with taxonomic experts. The checklist follows a similar format of previous liverwort and hornwort checklists by the authors, e.g., Java (Söderström et al. 2010). The checklist distinguishes between records that are based on specimens seen by the author(s) (reference in bold) and second-hand reports, e.g., citations of earlier publications (reference in normal type). All names used for the references are given after the taxon name with spelling variants/errors within quotation marks. Taxa are arranged in alphabetical order. Significantly, each accepted taxon is qualified using a four level ranking system that indicates our level of knowledge about a taxon. The coding convention is outlined in detail by von Konrat et al. (2010). Briefly, ? = Problem with the taxon name; * = Serious doubts about the value of the taxon; ** = Probably a good taxon (default value); *** = Accepted, a good taxon as currently understood.

This checklist covers the Lesser Sunda Islands based on reports from the literature that have been subdivided into 10 geographical units that correspond to geopolitical units. These include from west to east (Fig. 1) Bali, Lombok, Sumbawa, Sumba, Flores, Timor (separated in the Indonesian West Timor and the independent Timor-Leste), Barat Daya Islands, Tanimbar Islands, Kai Islands and Aru Islands.

## Types from Lesser Sunda Islands

Recently, there has been some debate whether the type information by Bonner (1962, 1963, 1965), as well as the other volumes of his “Index Hepaticarum”, can be accepted as lectotypes. Renner (2021) argued in favour for the volumes of Bonner (1962, 1963, 1965), including recommendations to improve typification practice, and Engel and Merrill (2019) argued that Bonner’s herbarium designations do not represent lectotypifications. Here we accept the typifications made by Bonner as they pertain to the taxa treated here, but we also provide alternative typifications if ruled against. In the latter cases, we instead “validate” Bonner’s typifications, yet causing no nomenclatural changes whichever view is taken.

It is not always clear if more than one collection exists for a possible type. McNeill (2014) recommends that in such a case a lectotype should be selected from known available material, but with a statement that it may be the only material, in which case a lectotypification would be superfluous. For such cases, we here follow McNeill’s “best practice” advice using the suggested phrase “lectotype here designated, if not a holotype”.

*Anastrophyllumintegerrimum* Steph., Sp. Hepat. (Stephani) 6: 107, 1917 (Stephani 1917). Originally described from “*Java* (Koorders leg), *Lombock*. (Elbert legit.)”. Lectotype (Bonner 1962): Java, 5000 pd., Koorders s.n. ex hb. Schinz, Zürich, G-00067196 (http://www.ville-ge.ch/musinfo/bd/cjb/chg/adetail.php?id=118174&lang=en). Specimen also annotated as “holotype” by J. Váňa in 1974. Note: If Bonner’s typification is rejected, we here designate the specimen as a new lectotype. The species was synonymized with *Gottscheliaschizopleura* by Grolle (1968).

*Chiloscyphuscommunis* Steph., Bull. Herb. Boissier (sér. 2) 7 (10): 839 [=Sp. Hepat. (Stephani) 3: 211], 1907 (Stephani 1907). Originally described from “*Java, Sumatra, Celebes, Nova Guinea, Timor, Samoa, Andaman Insulae, Queensland, Assam, Sikkim, Japan*, valde *communis.*”. Lectotype (Bonner 1963): Andamans, Port Blair, VIII.1890, E.H. Man, ex hb. Levier, G-00115054 (http://www.ville-ge.ch/musinfo/bd/cjb/chg/adetail.php?id=165692&lang=en). Specimen annotated as isotype by H. Inoue (undated). There are many specimens in G that Stephani may have seen, but apparently only this specimen corresponds to Bonner’s typification. The species was synonymized with *Chiloscyphusbescherellei* (=*Heteroscyphuscoalitus*) by Hattori (1966).

*Drepanolejeuneamoluccensis* Herzog, Ann. Bryol. 7: 88, 1934 (Herzog 1934). Originally described from “Molukken: Batjan, G. Sibella (Herb. H. B. Bog. n. 4103, leg. Roepke); Bali: Bratansee (Renner n. 331 ♀ u. ♂)”. Lectotype (here designated): Molukken, Batjan, G. Sibela, 1600–2000 m, W.K.J. Roepke s.n., JE-04002975 (https://herbarium.univie.ac.at/database/detail.php?ID=120304). Herzog noted “typus” on the specimen from Batjan while he noted “cotypus” on the specimen from Bali. The Bali material is issued in the exsiccatae “Hepaticae Selectae et Criticae (ed. Fr. Verdoorn) Ser. VIII (1965) 365” as “materia originalis” and should be present in several herbaria.

*Fimbrarialatifrons* Steph., Sp. Hepat. (Stephani) 6: 15, 1917 (Stephani 1917). Originally described from “*Lombock*. (Sunda Archipelagus.) (Elbert legit.)”. Lectotype (Long 2006: 227): [Indonesia] Lombok, Rindjani-Vulkangebirge, NNO Seite, Andjar Fluss, oberhalb; Monsun Hochwald, 1400–1530 m, humus Sand, 19.5.1909, J. Elbert 1388 (G-15225). Isolectotype: FH). [=G-00113144, http://www.ville-ge.ch/musinfo/bd/cjb/chg/adetail.php?id=144318&lang=en]. The species was synonymized with *Rebouliahemisphaerica* by Long (2006).

*Frullanialongispica* Steph., Sp. Hepat. (Stephani) 4: 454, 1911 (Stephani 1911). Originally described from “Insula Timor”. Type (Bonner 1965): Timor, 1100 m, IX 1897, Francis Newton s.n., G. However, there are two specimens in G with those collection data, G barcode 00069224 and 00265585. The former is annotated by Verdoorn in 1928 as “typica, .... species auton. non est!” (http://www.ville-ge.ch/musinfo/bd/cjb/chg/adetail.php?id=114964&lang=en), the latter does not have any annotation. Verdoorn never published any typification of *Frullanialongispica* but G00069224 is apparently the base for the synonymization with *Frullaniasquarrosa* in Verdoorn (1930) and is here designated as lectotype.

*Lepidozianewtonii* Steph., Sp. Hepat. (Stephani) 3: 623, 1909 (Stephani 1909). Originally described from “Insula Timor (Newton)”. Lectotype (here designated, if not holotype): Timor, 1100 m, Sept 1897, Francis Newton, Steph. herb. no. 126, G barcode 00069698 (http://www.ville-ge.ch/musinfo/bd/cjb/chg/adetail.php?id=135054&lang=en). There seems to be only one specimen in G that may be designated type, but we have not searched other herbaria and thus do not know if there are any possible type material elsewhere.

*Madothecaelbertii* Steph., Sp. Hepat. (Stephani) 6: 520, 1924 (Stephani 1924). Originally described from “India orientalis: Lombock (Elbert legit)”. Lectotype (Hattori 1969): Lombock, leg. leg. Dr. Elbert 2016, type of *Madothecaelbertii*, in herb. G (G barcode no. 00043932, http://www.ville-ge.ch/musinfo/bd/cjb/chg/adetail.php?id=130660&lang=en). The species was synonymized with Porellaacutifoliavar.lancifolia (= var. acutifolia) by Hattori (1976).

*Mastigobryumsumbavense* Steph., Hedwigia 25 (6): 236, 1886 (Stephani 1886). Originally described from “Insula Sumbawa, ad 4000’, Zollinger No. 3400b (Herb. Gottsche)”. Lectotype (Mizutani 1967, ‘type’): Insula Sumbava, Montis Batu, Alt. 4000 ft., Lante ad arbores, Zollinger 3400b, Steph. herb. no. 10769, G barcode 00066907. If Mizutani’s type specification is rejected we here designate the mentioned specimen as new lectotype (if it is not a holotype).

## List of accepted species and infraspecific taxa

### Anthocerotophyta

***Folioceros***

*** *Foliocerosfuciformis* (Mont.) D.C.Bharadwaj **Bali**: **Schiffner 1955** as *Aspiromitusfalsinervius*.

### Marchantiophyta

***Acanthocoleus***

*** *Acanthocoleusjavanicus* (Steph.) Kruijt. **Bali**: **Wilson et al. 2007**; **Heinrichs et al. 2012**; **Dong et al. 2013**; Haerida 2017.

***Acrolejeunea***

*** *Acrolejeuneaaulacophora* (Mont.) Steph. **Lesser Sunda Is.**: **Bali**: **Hegewald and van Zanten 1986**; Haerida 2017.

*** *Acrolejeuneafertilis* (Reinw., Blume et Nees) Schiffn. **Bali**: **Gradstein 1975**; **Wilson et al. 2004**; **Forrest et al. 2006**; **Wilson et al. 2007**; **Heinrichs et al. 2012**; **Czumay et al. 2013**; **Dong et al. 2013**; **Heinrichs et al. 2014a**; **Heinrichs et al. 2014b**; **Schäfer-Verwimp et al. 2014**; **Bechteler et al. 2016a**; **Bechteler et al. 2016b**; Haerida 2017. **Tanimbar Islands**: **Gradstein 1975**. **Timor**: **Gradstein 1975**.

***Aneura***

*** *Aneuramaxima* (Schiffn.) Steph. **Bali**: **Haerida 2017**.

*** *Aneurapinguis* (L.) Dumort. **Bali**: Haerida et al. 2015; **Haerida 2017**.

***Asterella***

*** *Asterellablumeana* (Nees) Kachroo **Lombok**: **Long 2006**.

*** *Asterellavulcanica* (Schiffn.) Kachroo et Bapna **Bali**: Haerida et al. 2015; **Haerida 2017**.

***Bazzania***

*** *Bazzanialongicaulis* (Sande Lac.) Schiffn. **Sumba**: **Haerida et al. 2020**.

* ^1^*Bazzaniasumbavensis* (Gottsche ex Steph.) Steph. **Sumbawa**: **Lectotype** of *Mastigobryumsumbavense*, **Stephani 1886** as *Mastigobryumsumbavense*, **Schiffner 1898**; **Stephani 1908** as *Mastigobryumsumbavense*, Bonner 1963; Grolle 1966; **Mizutani 1967**; Tixier 1974; Kamimura 1975; Miller et al. 1983; Miller et al. 1983 as *Mastigobryumsumbavense*, **Geissler and Bischler 1985** as *Mastigobryumsumbavense*, Long and Grolle 1990; **Sharma and Srivastava 1993**; Bapna and Kachroo 2000.

***Cheilolejeunea***

** *Cheilolejeuneaceylanica* (Gottsche) R.M.Schust. et Kachroo **Sumba**: **Haerida et al. 2020**.

*** *Cheilolejeuneatrapezia* (Nees) Kachroo et R.M.Schust. **Bali**: **Haerida 2017**. **Sumba**: **Haerida et al. 2020**.

***Chiastocaulon***

*** *Chiastocaulondendroides* (Nees) Carl. **Bali**: **So 2001** as *Plagiochiladendroides*.

*** *Chiastocaulonoppositum* (Reinw., Blume et Nees) S.D.F.Patzak, M.A.M.Renner, Schäf.-Verw. et Heinrichs. **Lesser Sunda Is.**: Pócs et al. 2011 as *Plagiochilionoppositum*. **Bali**: **Hegewald and van Zanten 1986** as *Plagiochilionoppositum*, **Groth and Heinrichs 2005** as *Plagiochilionoppositum*, Haerida et al. 2015 as *Plagiochilionoppositum*, **Patzak et al. 2016**; **Renner et al. 2016**; Haerida 2017; **Renner et al. 2017**. **Sumbawa**: **Sande Lacoste 1864** as *Plagiochilaopposita*, **Schiffner 1898** as *Plagiochilaopposita*, Miller et al. 1983 as *Plagiochilionoppositum*.

***Cololejeunea***

*** *Cololejeuneaangustiflora* (Steph.) Mizut. **Bali**: **Haerida 2017**.

*** *Cololejeuneaappressa* (A.Evans) Benedix. **Bali**: **Haerida 2017**.

** *Cololejeuneagottschei* (Steph.) Pandé, K.P.Srivast. et Ahmad **Bali**: **Haerida 2017**.

*** *Cololejeuneamacounii* (Spruce) A.Evans **Bali**: **Haerida 2017**.

*** *Cololejeuneaobliqua* (Nees et Mont.) Schiffn. **Bali**: **Benedix 1953** as *Cololejeuneanymannii*, Tixier 1962 as *Cololejeuneanymannii*, Tixier 1973 as *Cololejeuneanymannii*, Miller et al. 1983 as *Cololejeuneanymannii*.

*** *Cololejeuneaocelloides* (Horik.) Mizut. **Bali**: Tixier 1962 as Cololejeunealeonidensvar.saccata.

** *Cololejeuneasubfloccosa* Mizut. **Bali**: **Haerida 2017**.

** *Cololejeuneatriapiculata* (Herzog) Tixier **Bali**: **Haerida 2017**.

*** *Cololejeuneatrichomanis* (Gottsche) Besch. **Bali**: **Haerida 2017** as *Cololejeuneagoebelii*.

***Colura***

** ^2^*Coluraleratii* (Steph.) Steph. **Bali**: **Eggers et al. 1998**; Haerida 2017. **Flores**: **Jovet-Ast 1967** as *Coluraapiculata*, Eggers et al. 1998.

***Diplasiolejeunea***

*** *Diplasiolejeuneacavifolia* Steph. **Bali**: **Schäfer-Verwimp 2006**^3^; Haerida 2017; Siregar et al. 2020.

***Drepanolejeunea***

*** *Drepanolejeunealevicornua* Steph. **Sumba**: **Haerida et al. 2020**.

** *Drepanolejeuneamoluccensis* Herzog. **Bali**: **Syntype**, **Herzog 1934**; Verdoorn 1935; Tixier 1979.

*** *Drepanolejeuneapentadactyla* (Mont.) Steph. **Bali**: **Haerida 2017**.

***Dumortiera***

*** *Dumortierahirsuta* (Sw.) Nees **Bali**: Haerida et al. 2015; **Haerida 2017**.

***Fossombronia***

*** *Fossombroniahimalayensis* Kashyap. **Bali**: **Krayesky et al. 2005**.

***Frullania***

** *Frullaniaapiculata* (Reinw., Blume et Nees) Nees **Sumba**: **Haerida et al. 2020**.

*** *Frullaniaericoides* (Nees) Mont. **Bali**: **Hegewald and van Zanten 1986**; Haerida 2017. **Lombok**: **Verdoorn 1934b** as *Frullaniasquarrosa*. **Timor**: **Type** of *Frullanialongispica*, **Stephani 1911** as *Frullanialongispica*, **Bonner 1965** as *Frullanialongispica*.

*** *Frullaniagaudichaudii* (Nees et Mont.) Nees et Mont. **Bali**: **Haerida 2015**; **Haerida 2017**.

*** *Frullaniagracilis* (Reinw., Blume et Nees) Nees **Bali**: **Haerida 2015**; **Haerida 2017**; Rosyanti et al. 2018.

** Frullaniaintermedia(Reinw., Blume et Nees)Neessubsp.intermedia. **Aru Islands**: **Hattori 1980**.

*** *Frullaniajunghuhniana* Gottsche. **Bali**: **Haerida 2015**; **Haerida 2017**.

*** *Frullaniameyeniana* Lindenb. **Bali**: **Haerida 2015**; **Haerida 2017**; Rosyanti et al. 2018.

*** ^4^*Frullaniamoniliata* (Reinw., Blume et Nees) Mont. **Bali**: **Haerida 2015**; **Haerida 2017**. **Sumba**: **Haerida et al. 2020**. **Sumbawa**: **Sande Lacoste 1856**; **Schiffner 1898**; Verdoorn 1930 as Frullaniamoniliatasubsp.breviramea, **Hattori 1975** as Frullaniatamariscivar.breviramea.

*** *Frullanianodulosa* (Reinw., Blume et Nees) Nees **Aru Islands**: **Mitten 1885** as *Frullaniasecundiflora*, **Schiffner 1898** as *Frullaniasecundiflora*. **Flores**: **Hattori 1975**. **Kai Islands**: **Mitten 1885** as *Frullaniasecundiflora*, **Schiffner 1898** as *Frullaniasecundiflora*, Miller et al. 1983. **Sumba**: **Verdoorn 1930**; **Haerida et al. 2020**. **Sumbawa**^5^: Hattori 1951; Swanson and Miller 1969; Miller et al. 1983. **Tanimbar Islands**: **Hattori 1980** as Frullanianodulosavar.nodulosa. **Timor**: **Hattori 1980** as Frullanianodulosavar.nodulosa.

*** *Frullaniaornithocephala* (Reinw., Blume et Nees) Nees **Bali**: **Haerida 2015**; **Haerida 2017**. **Lombok**: **Verdoorn 1934b**.

*** *Frullaniario-janeirensis* (Raddi) Ångstr. **Bali**: **Hegewald and van Zanten 1986**; Enroth 1991 as *Frullaniaafricana*, **Haerida 2017**.

*** *Frullaniaserrata* Gottsche **Lombok**: **Verdoorn 1934b**.

*** *Frullaniaternatensis* Gottsche **Bali**: **Haerida 2015**; Haerida 2017.

** — var. non-appendiculata S.Hatt. **Bali**: **Hegewald and van Zanten 1986**.

* ^6^*Frullaniatricarinata* Sande Lac. **Bali**: **Hegewald and van Zanten 1986** as *Frullania* ‘*tricapinata*’, Haerida 2017; Winter and Schäfer-Verwimp 2020.

*** *Frullaniatrichodes* Mitt. **Kai Islands**: **Verdoorn 1937** as *Frullaniatenuicaulis*.

***Gottschelia***

*** *Gottscheliaschizopleura* (Spruce) Grolle **Lesser Sunda Is.**: Váňa and Piippo 1989b; Váňa 1991b. **Lombok**: **Syntype** of *Anastrophyllumintegerrimum*, **Stephani 1917** as *Anastrophyllumintegerrimum*, Bonner 1962 as *Anastrophyllumintegerrimum*, Grolle 1968; Miller et al. 1983 as *Anastrophyllumintegerrimum*.

***Herbertus***

** *Herbertusceylanicus* (Steph.) Abeyw. **Flores**: **Juslén 2006**.

*** *Herbertusdicranus* (Gottsche, Lindenb. et Nees) Trevis. **Bali**: **Juslén 2006**; Váňa et al. 2014.

** *Herbertuslongispinus* J.B.Jack et Steph. **Flores**: **Juslén 2006**.

** *Herbertusramosus* (Steph.) H.A.Mill. **Bali**: **Hegewald and van Zanten 1986**; Haerida 2017.

*** *Herbertussendtneri* (Nees) Lindb. **Lesser Sunda Is.**: **Juslén 2006** as *Herbertusarmitanus*.

***Heteroscyphus***

*** *Heteroscyphusargutus* (Reinw., Blume et Nees) Schiffn. **Aru Islands**: **Mitten 1885** as *Chiloscyphusargutus*, **Schiffner 1898** as *Chiloscyphusargutus*. **Bali**: Haerida et al. 2015; **Haerida 2017**.

*** *Heteroscyphusaselliformis* (Reinw., Blume et Nees) Schiffn. **Bali**: **Haerida 2017**. **Sumbawa**: **Zollinger 1855** as *Chiloscyphusaselliformis*, **Sande Lacoste 1856** as *Chiloscyphusaselliformis*, **Schiffner 1898** as *Chiloscyphusaselliformis*, Schiffner 1900 as *Chiloscyphusaselliformis*, Miller et al. 1983; Piippo 1985; Piippo 1989b; Yamada and Hayashi 2003.

*** *Heteroscyphuscoalitus* (Hook.) Schiffn. **Bali**: **Hegewald and van Zanten 1986** as *Chiloscyphuscoalitus*, Srivastava and Srivastava 2002; Haerida et al. 2015; **Haerida 2017**. **Sumba**: **Haerida et al. 2020**. **Timor**: **Syntype** of *Chiloscyphuscommunis*, Stephani 1907 as *Chiloscyphuscommunis*, Hattori 1951 as *Heteroscyphuscommunis*, Miller 1968 as *Chiloscyphuscommunis*, Swanson and Miller 1969 as *Chiloscyphuscommunis*, Piippo 1993. Timor-Leste: Piippo 1985; Piippo 1989b.

*** *Heteroscyphussplendens* (Lehm. et Lindenb.) Grolle **Sumba**^7^: Miller et al. 1983 as *Heteroscyphusdecurrens*. **Sumbawa**: **Zollinger 1855** as *Chiloscyphusdecurrens*, **Sande Lacoste 1856** as *Chiloscyphusdecurrens*, **Schiffner 1898** as *Chiloscyphusdecurrens*, Pócs 1971 as *Heteroscyphusdecurrens*.

***Jackiella***

*** *Jackiellajavanica* Schiffn. **Bali**: **Haerida 2017**.

***Lejeunea***

*** *Lejeuneaalata* Gottsche **Sumba**: **Haerida et al. 2020**.

*** *Lejeuneaapiculata* Sande Lac. **Bali**: **Haerida 2017**.

*** *Lejeuneamimula* Hürl. **Bali**: **Wilson et al. 2004**; **Gradstein et al. 2006**; **Wilson et al. 2007**; **Heinrichs et al. 2012**; **Czumay et al. 2013**; **Dong et al. 2013**; **Heinrichs et al. 2014a**; **Heinrichs et al. 2014b**; Haerida 2017.

***Lepidozia***

* *Lepidozianewtonii* Steph. **Timor**: **Type**, **Stephani 1909**.

***Leptolejeunea***

*** *Leptolejeuneaelliptica* (Lehm. et Lindenb.) Besch. **Sumba**: **Haerida et al. 2020**.

*** *Leptolejeuneaepiphylla* (Mitt.) Steph. **Bali**: **Haerida 2017**.

** *Leptolejeuneafoliicola* Steph. **Bali**: **Eggers et al. 1998**; **Bechteler et al. 2016c**; Haerida 2017; **Shu and Zhu 2019**.

* *Leptolejeuneamassartiana* Schiffn. ex Herzog **Bali**: **Eggers et al. 1998**; Haerida 2017.

*** *Leptolejeuneasubacuta* Steph. ex A.Evans **Bali**: **Haerida 2017**.

***Lopholejeunea***

*** *Lopholejeuneaeulopha* (Taylor) Schiffn. **Bali**: **Zhu and Gradstein 2005**; Haerida et al. 2010; Haerida 2017; Siregar et al. 2020.

*** *Lopholejeuneahorticola* Schiffn. **Bali**: **Zhu and Gradstein 2005**; Haerida et al. 2010; Siregar et al. 2014; Siregar 2015; Pócs and Chantanaorrapint 2016; Haerida 2017.

*** *Lopholejeuneanigricans* (Lindenb.) Schiffn. **Bali**: **Haerida 2017**. **Sumba**: **Haerida et al. 2020**.

*** *Lopholejeunearecurvata* Mizut. **Bali**: **Zhu and Gradstein 2005**; Haerida et al. 2010; Haerida 2017.

*** *Lopholejeuneasubfusca* (Nees) Schiffn. **Lesser Sunda Is.**: Pócs et al. 1967. **Aru Islands**: **Mitten 1885** as *Lejeuneasubfusca*, **Schiffner 1898** as Lopholejeuneasagranavar.subfusca. **Bali**: **Zhu and Gradstein 2005**; Haerida 2009; Haerida et al. 2010; Siregar et al. 2014; Siregar 2015; **Haerida 2017**; Rosyanti et al. 2018; Siregar et al. 2020.

*** *Lopholejeuneazollingeri* (Steph.) Schiffn. **Bali**: **Haerida 2017**.

***Marchantia***

*** *Marchantiaacaulis* Steph. **Bali**: Haerida et al. 2015; **Haerida 2017**.

*** *Marchantiaemarginata* Reinw., Blume et Nees **Bali**: Siregar et al. 2013; Haerida et al. 2015; Siregar 2015; **Haerida 2017**; Ginting and Batubara 2019.

*** — subsp. emarginata. **Bali**: **Bischler-Causse 1989**; Bischler and Piippo 1991. **Flores**: **Bischler-Causse 1989**; Bischler and Piippo 1991.

*** *Marchantiageminata* Reinw., Blume et Nees **Bali**: Haerida et al. 2015; **Haerida 2017**. **Flores**: **Bischler-Causse 1989**.

*** *Marchantiatreubii* Schiffn. **Lesser Sunda Is.**: Siregar et al. 2013; Siregar 2015. **Bali**: **Haerida 2017**. **Flores**: **Bischler-Causse 1989**. **Lombok**: **Bischler-Causse 1989**. **Timor**: **Stephani 1899**; **Bischler-Causse 1989**.

***Metalejeunea***

*** *Metalejeuneacucullata* (Reinw., Blume et Nees) Grolle **Bali**: **Bechteler et al. 2016b**.

***Metzgeria***

*** *Metzgeriaciliata* Raddi **Bali**: **Haerida 2017**.

*** *Metzgeriaconsanguinea* Schiffn. **Sumba**: **Haerida et al. 2020**.

* *Metzgeriafoliicola* Schiffn. **Flores**: **So 2003b**.

*** *Metzgerialindbergii* Schiffn. **Bali**: **Haerida 2017**. **Flores**: **So 2003b**.

***Pallavicinia***

*** *Pallavicinialyellii* (Hook.) Gray **Bali**: **Haerida 2017**.

***Plagiochila***

*** *Plagiochilabantamensis* (Reinw., Blume et Nees) Mont. **Bali**: **So 2001**.

*** *Plagiochilafrondescens* (Nees) Lindenb. **Bali**: **Inoue 1984**; Piippo 1989a; **Patzak et al. 2016**; **Renner et al. 2017**.

*** *Plagiochilajavanica* (Sw.) Nees et Mont. **Bali**: **Inoue 1984**; **Hegewald and van Zanten 1986**; Piippo 1989a; Haerida 2017.

*** *Plagiochilajunghuhniana* Sande Lac. **Bali**: **So 2001**.

* ^8^*Plagiochilakuhliana* Sande Lac. **Bali**: **Inoue 1984**.

** *Plagiochilamassalongoana* Schiffn. **Bali**: **Inoue 1984**.

*** *Plagiochilaobtusa* Lindenb. **Bali**: **So 2001**.

*** *Plagiochilaparvifolia* Lindenb. **Bali**: **So 2001**.

** *Plagiochilapropinqua* Sande Lac. **Bali**: **Inoue 1984**; Inoue 1989; Piippo 1989a; Piippo and Tan 1992; Grolle and So 1999b.

*** *Plagiochilarenitens* (Nees) Lindenb. **Bali**: **Inoue 1984**; Inoue 1989; Piippo 1989a.

*** *Plagiochilasalacensis* Gottsche **Bali**: **Carl 1931** as *Plagiochilajackii*, **Hegewald and van Zanten 1986**; **Grolle and So 1999a**; **So 2001**; Siregar 2015; Haerida 2017; Siregar et al. 2018.

*** *Plagiochilasciophila* Nees **Bali**: **Inoue 1984**; Inoue 1989; Piippo 1989a; Enroth 1991; Siregar 2015; Siregar et al. 2018.

** *Plagiochilasemidecurrens* (Lehm. et Lindenb.) Lindenb. **Bali**: **So 2001**.

** *Plagiochilaspathulifolia* Mitt. **Bali**: **Inoue 1984**; Inoue 1989.

*** *Plagiochilateysmannii* Sande Lac. **Bali**: **Hegewald and van Zanten 1986**; So and Grolle 1999; Haerida 2017. **Sumba**: **Haerida et al. 2020**.

***Pleurozia***

*** *Pleuroziagigantea* (F.Weber) Lindb. **Flores**: **Thiers 1993**.

***Porella***

** *Porellaacutifolia* (Lehm. et Lindenb.) Trevis. **Bali**: **Hegewald and van Zanten 1986**; Haerida 2017. **Lombok**: Miller et al. 1983.

** — var. acutifolia. **Lombok**: **Lectotype** of *Madothecaelbertii*, **Stephani 1924** as *Madothecaelbertii*, **Hattori 1969** as Porellaacutifoliavar.elbertii.

***Ptychanthus***

*** *Ptychanthusstriatus* (Lehm. et Lindenb.) Nees **Bali**: **Haerida 2017**. **Lombok**: **Verdoorn 1934a**; Miller et al. 1983. **Sumba**: **Haerida et al. 2020**.

***Radula***

** *Radulaacuminata* Steph. **Bali**: **Haerida 2017**.

*** *Radulacampanigera* Mont. **Bali**: **Haerida 2017**.

** *Radulajavanica* Gottsche **Bali**: **Haerida 2017**. **Sumba**: **Haerida et al. 2020**.

* *Radulamultiflora* Gottsche ex Schiffn. **Aru Islands**: **Schiffner 1898**. **Bali**: **Hegewald and van Zanten 1986**; Haerida 2017.

* *Radulapinnulata* Mitt. **Aru Islands**: **Mitten 1885**; **Schiffner 1898**.

*** *Radulaventricosa* Steph. **Bali**: **Haerida 2017**.

***Reboulia***

*** *Rebouliahemisphaerica* (L.) Raddi **Bali**: Haerida et al. 2015; **Haerida 2017**. **Lombok**: **Lectotype** of *Fimbrarialatifrons*, **Stephani 1917** as ‘*Fimbriaria’ latifrons*. **Bonner 1965** as ‘*Fimbriaria’ latifrons*, **Long 2006**.

***Riccardia***

** *Riccardiacrenulata* Schiffn. **Bali**: **Schiffner 1955** as *Riccardiatenuicostata*. **Sumba**: **Haerida et al. 2020** as *Aneuracrenulata*.

***Riccia***

*** *Ricciabillardierei* Mont. et Nees **Bali**: Jovet-Ast 2000; **Jovet-Ast 2003**.

*** *Ricciacruciata* Kashyap. **Bali**: **Jovet-Ast 2003**.

*** *Ricciadiscolor* Lehm. et Lindenb. **Bali**: **Jovet-Ast 2003**.

*** *Ricciajunghuhniana* Nees et Lindenb. **Bali**: **Jovet-Ast 2003**; **Haerida 2017**.

*** *Ricciamangalorica* Ahmad ex Jovet-Ast **Bali**: **Jovet-Ast 2003** as *Ricciamangalorica*.

***Scapania***

*** *Scapaniajavanica* Gottsche **Bali**: **Blockeel et al. 2009**; Haerida 2017.

***Schiffneriolejeunea***

*** *Schiffneriolejeuneatumida* (Nees) Gradst. **Bali**: **Wilson et al. 2004**.

*** — var. haskarliana (Gottsche) Gradst. et Terken. **Bali**: **Wilson et al. 2007**.

***Schistochila***

*** *Schistochilaaligera* (Nees et Blume) J.B.Jack et Steph. **Bali**: **So 2003a**. **Flores**: **So 2003a**.

*** *Schistochilablumei* (Nees) Trevis. **Bali**: **So 2003a**.

***Solenostoma***

*** *Solenostomatetragonum* (Lindenb.) R.M.Schust. ex Váňa et D.G.Long **Bali**: **Váňa 1972**, 1973, 1975, 1991a all as *Jungermanniatetragona*, Miller et al. 1983 as *Jungermanniatetragona*, Váňa and Piippo 1989a as *Jungermanniatetragona*, Bapna and Kachroo 2000 as *Jungermanniatetragona*, Srivastava and Sharma 2000 as *Jungermanniatetragona*, Easa 2003 as *Jungermanniatetragona*.

*** *Solenostomatruncatum* (Nees) R.M.Schust. ex Váňa et D.G.Long **Bali**: **Váňa and Piippo 1989a** as *Jungermanniatruncata*, Váňa 1991a as *Jungermanniatruncata*, Bapna and Kachroo 2000 as *Jungermanniatruncata*.

***Spruceanthus***

*** *Spruceanthuspolymorphus* (Sande Lac.) Verd. **Aru Islands**: **Mitten 1885** as *Phragmicomapolymorpha*, **Schiffner 1898** as *Thysananthuspolymorphus*. **Sumba**: **Haerida et al. 2020**.

*** *Spruceanthussemirepandus* (Nees) Verd. **Lombok**: **Verdoorn 1934a**; Jovet-Ast and Schmid 1958; Kitagawa 1981.

***Thysananthus***

*** *Thysananthushumilis* (Gottsche) Sukkharak et Gradst. **Bali**: **Sukkharak and Gradstein 2014** as *Mastigolejeuneahumilis*. **Sumba**: **Haerida et al. 2020** as *Mastigolejeuneahumilis*.

*** *Thysananthusligulatus* (Lehm. et Lindenb.) Sukkharak et Gradst. **Sumba**: **Haerida et al. 2020** as *Mastigolejeunealigulata*.

*** *Thysananthusspathulistipus* (Reinw., Blume et Nees) Lindenb. **Bali**: **Wilson et al. 2007**; Haerida et al. 2010; **Sukkharak 2011**; **Heinrichs et al. 2012**; **Czumay et al. 2013**; **Dong et al. 2013**; **Heinrichs et al. 2014a**; **Heinrichs et al. 2014b**; **Schäfer-Verwimp et al. 2014**; Siregar et al. 2014; Siregar 2015; **Sukkharak 2015**; **Bechteler et al. 2016a**; **Bechteler et al. 2016b**; **Haerida 2017**; Siregar et al. 2017; Siregar et al. 2020. **Kai Islands**: **Verdoorn 1937**. **Sumbawa**: **Zollinger 1855**; **Schiffner 1898**; **Verdoorn 1934a**; Swanson and Miller 1969; Miller et al. 1983; Haerida et al. 2010; Siregar et al. 2014; Siregar 2015; Siregar et al. 2017.

*** *Thysananthusvirens* Ångstr. **Bali**: **Sukkharak and Gradstein 2014b** as *Mastigolejeuneavirens*, Siregar et al. 2020 as *Mastigolejeuneavirens*.

***Wiesnerella***

*** *Wiesnerelladenudata* (Mitt.) Steph. **Bali**: **Haerida 2017**.

#### Taxa of unclear affinity

A couple of taxa are published from Lesser Sunda Islands as varieties of species synonymized under other names, without transferring or synonymizing the variety. We have not been able to trace any specimen that they may be based on and, thus, not been able to refer them to any valid taxon.

***Chiloscyphus***

? ChiloscyphuszollingeriGottschevar.subintegerrimus Schiffn. **Bali**: **Schiffner 1955**. Note: *Chiloscyphuszollingeri* Gottsche is now Heteroscyphuszollingeri but we are not sure if var. subintegerrimus also belongs to that species and is worth recognizing.

***Riccardia***

? RiccardiaplatycladaSchiffn.var.leiomitra Schiffn. **Bali**: **Schiffner 1955**. Note: *Riccardiaplatyclada* Schiffn. is a synonym of R.graeffei but it is unclear where var. leiomitra from Java belongs (Söderström et al. 2010).

#### Taxa reported but doubtfully occurring in Lesser Sunda Islands

### Marchantiophyta

***Bazzania***

** *Bazzaniaceylanica* (Mitt.) Steph. **Lesser Sunda Is.**: Miller et al. 1983. Note: The report by Miller et al. is unclear and it may be that they meant some of the Greater Sunda Islands. It is widespread in SE Asia, so its presence is not unlikely.

*** *Bazzaniaerosa* (Reinw., Blume et Nees) Trevis. **Lesser Sunda Is.**: Miller et al. 1983. Note: The report by Miller et al. is unclear and it may be that they meant some of the Greater Sunda Islands. It is widespread in SE Asia so its presence is not unlikely.

*** *Bazzaniatridens* (Reinw., Blume et Nees) Trevis. **Lesser Sunda Is.**: Miller et al. 1983. **Sumbawa**: Pócs 1971. Note: We are not aware of any first hand report from Lesser Sunda Islands but it is common in SE Asia so its presence on some of the islands is not unlikely.

***Ceratolejeunea***

*** Ceratolejeuneacf.papuliflora Steph. **Sumba**: Haerida et al. 2020. Note: The species was only reported with doubt. It is otherwise not reported outside Africa, but it occurs on the Western Indian Ocean Islands and may perhaps also occur in SE Asia.

***Cololejeunea***

*** Cololejeuneacf.lanciloba Steph. **Bali**: Haerida 2017. Note: The species was only reported with doubt. However, it is widespread in SE Asia and its occurrence in the area is not unlikely.

** Cololejeuneacf.serrulata Steph. **Bali**: Haerida 2017. Note: The species was only reported with doubt. It occurs on other Islands in SE Asia so its presence on the Lesser Sunda Islands is not unlikely.

***Colura***

*** *Coluraari* (Steph.) Steph. **Lesser Sunda Is.**: Miller et al. 1983. Note: The report by Miller et al. is unclear and it may be that they meant some of the Greater Sunda Islands. It is widespread in SE Asia so its presence is not unlikely.

** *Coluraimperfecta* Steph. **Lesser Sunda Is.**: Miller et al. 1983. Note: The report by Miller et al. is unclear and it may be that they meant some of the Greater Sunda Islands. It is widespread in SE Asia so its presence is not unlikely.

***Conoscyphus***

*** *Conoscyphustrapezioides* (Sande Lac.) Schiffn. **Lesser Sunda Is.**: Miller et al. 1983 as *Chiloscyphustrapezioides*. Note: The report by Miller et al. is unclear and it may be that they meant some of the Greater Sunda Islands. It is widespread in SE Asia so its presence is not unlikely.

***Drepanolejeunea***

*** *Drepanolejeuneaternatensis* (Gottsche) Schiffn. **Lesser Sunda Is.**: Miller et al. 1983. Note: The report by Miller et al. is unclear and it may be that they meant some of the Greater Sunda Islands. It is widespread in SE Asia so its presence is not unlikely.

***Radula***

*** *Radulacomplanata* (L.) Dumort. **Bali**: **Haerida 2017.** Note: A mainly boreal species that have its closest known occurrences in Himalaya. The report was erroneously published without a ‘cf’ as the identification was only preliminary (I. Haerida).

***Targionia***

*** *Targioniahypophylla* L. **Bali**: Haerida 2017. Note: The species was only reported with doubt. It is widespread but as the relation to other taxa remains unclear, its distribution is also unclear.

#### Taxa reported but rejected from Lesser Sunda Islands

### Marchantiophyta

***Ceratolejeunea***

*** *Ceratolejeuneaceratantha* (Nees et Mont.) Schiffn. **Sumbawa**: **Sande Lacoste 1864** as *Lejeuneaceratantha*, **Schiffner 1898**. Note: This is a Neotropical taxon and the old reports from Asia must be rejected.

***Frullania***

* *Frullanialudoviciae* Steph. **Sumbawa**: Miller et al. 1983 (with a ‘?’). Note: Hattori (1986) is rejecting earlier records outside New Caledonia as based on erroneous synonymization of *F.tenuirostris*.

***Thysananthus***

*** *Thysananthusauriculatus* (Wilson et Hook.) Sukkharak et Gradst. **Bali**: **Wilson et al. 2004** as *Mastigolejeuneaauriculata*, **Wilson et al. 2007** as *Mastigolejeuneaauriculata*, **Ye and Zhu 2018**. Note: Sukkharak and Gradstein (2014) rejects all report of this American-African taxon from SE Asia as *Thysananthushumilis*.

#### Synonyms

*Anastrophyllumintegerrimum* Steph. = *Gottscheliaschizopleura*

*Aneuracrenulata* (Schiffn.) Steph. ≡ *Riccardiacrenulata*

*Aspiromitusfalsinervius* (Lindenb. ex Meissner) Steph. = *Foliocerosfuciformis*

*Chiloscyphusargutus* (Reinw., Blume et Nees) Nees ≡ *Heteroscyphusargutus*

*Chiloscyphusaselliformis* (Reinw., Blume et Nees) Nees ≡ *Heteroscyphusaselliformis*

*Chiloscyphuscoalitus* (Hook.) Nees ≡ *Heteroscyphuscoalitus*

*Chiloscyphuscommunis* Steph. = *Heteroscyphuscoalitus*

*Chiloscyphusdecurrens* (Reinw., Blume et Nees) Nees = *Heteroscyphussplendens*

*Chiloscyphustrapezioides* Sande Lac. ≡ *Conoscyphustrapezioides*

*Cololejeuneagoebelii* (Gottsche ex Schiffn.) Schiffn. = *Cololejeuneatrichomanis*

Cololejeunealeonidensvar.saccata Benedix = *Cololejeuneaocelloides*

*Cololejeuneanymannii* (Steph.) Benedix = *Cololejeuneaobliqua*

*Coluraapiculata* Steph. = *Coluraleratii*

*Fimbrarialatifrons* Steph. = *Rebouliahemisphaerica*

*Frullaniaafricana* Steph. = *Frullaniario-janeirensis*

*Frullanialongispica* Steph. = *Frullaniaericoides*

Frullaniamoniliatasubsp.breviramea (Steph.) Verd. = *Frullaniamoniliata*

*Frullaniasecundiflora* Mont. = *Frullanianodulosa*

*Frullaniasquarrosa* (Mont.) Nees = *Frullaniaericoides*

Frullaniatamariscivar.breviramea (Steph.) S.Hatt. = *Frullaniamoniliata*

*Frullaniatenuicaulis* Mitt. = *Frullaniatrichodes*

*Herbertusarmitanus* (Steph.) H.A.Mill. = *Herbertussendtneri*

*Heteroscyphuscommunis* (Steph.) Schiffn. = *Heteroscyphuscoalitus*

*Heteroscyphusdecurrens* (Nees) Schiffn. = *Heteroscyphussplendens*

*Jungermanniatetragona* Lindenb. ≡ *Solenostomatetragonum*

*Jungermanniatruncata* Nees ≡ *Solenostomatruncatum*

*Lejeuneaceratantha* Nees et Mont. ≡ *Ceratolejeuneaceratantha*

*Lejeuneasubfusca* (Nees) Nees et Mont. ≡ *Lopholejeuneasubfusca*

*Lopholejeuneasagrana* var. *β subfusca* (Nees) Schiffn. ≡ *Lopholejeuneasubfusca*

*Madothecaelbertii* Steph. = Porellaacutifoliavar.acutifolia

*Mastigobryumsumbavense* Gottsche ex Steph. ≡ *Bazzaniasumbavensis*

*Mastigolejeuneaauriculata* (Wilson et Hook.) Steph. ≡ *Thysananthusauriculatus*

*Mastigolejeuneahumilis* (Gottsche) Schiffn. ≡ *Thysananthushumilis*

*Mastigolejeunealigulata* (Lehm. et Lindenb.) Schiffn. ≡ *Thysananthusligulatus*

*Mastigolejeuneavirens* (Ångstr.) Steph. ≡ *Thysananthusvirens*

*Phragmicomapolymorpha* Sande Lac. ≡ *Spruceanthuspolymorphus*

*Plagiochiladendroides* (Nees) Lindenb. ≡ *Chiastocaulondendroides*

*Plagiochilajackii* Schiffn. nom. illeg. = *Plagiochilasalacensis*

*Plagiochilaopposita* (Reinw., Blume et Nees) Lindenb. ≡ *Chiastocaulonoppositum*

*Plagiochilionoppositum* (Reinw., Blume et Nees) S.Hatt. ≡ *Chiastocaulonoppositum*

Porellaacutifoliavar.elbertii (Steph.) S.Hatt. = Porellaacutifoliavar.acutifolia

*Riccardiatenuicostata* Schiffn. nom. illeg. = *Riccardiacrenulata*

*Riccardiatenuicostata* Schiffn. = *Riccardiainconspicua*

*Ricciamangalorica* Ahmad nom. inval. ≡ *Ricciamangalorica*

*Thysananthuspolymorphus* (Sande Lac.) Schiffn. ≡ *Spruceanthuspolymorphus*

## Footnotes

Bazzania sumbavensis is possibly conspecific with Bazzania japonica (Söderström et al. 2010).

Colura leratii was omitted in Söderström et al. (2016) as the basionym was erroneously considered invalid.

The collecting data for the report of Diplasiolejeunea cavifolia in Schäfer-Verwimp (2006) is “Nordbali, Distr. Tabanan, Bedugul, Aufstieg im Nebelwald zum Gunung Catur am Bratansee, epiphyll an Pandanus sp.; 1260 m, 30. Mai 1995, leg. Schäfer-Verwimp & Verwimp, det. Schäfer-Verwimp (Hb Schäfer-Verwimp No. 16750)” (A. Schäfer-Verwimp in litt. 2021).

Frullania moniliata is a species complex (Vilnet et al. 2014) in sect. Thyopsiella.

We do not know of any first hand report of Frullania nodulosa from Sumbawa.

Frullania tricarinata is possibly conspecific with Frullania hypoleuca (Söderström et al. 2010).

We do not know of any first hand report of Heteroscyphus splendens from Sumba. It may be an error for Sumbawa.

Plagiochila kuhliana is possibly conspecific with Plagiochila sciophila (Söderström et al. 2010).
